# Omental pregnancy: case report and review of literature

**DOI:** 10.11604/pamj.2014.19.244.3661

**Published:** 2014-11-05

**Authors:** Antonio Maiorana, Domenico Incandela, Laura Giambanco, Walter Alio, Luigi Alio

**Affiliations:** 1 Gynecology and Obstetrics Unit, Palermo Civic Hospital and National Center of Clinical Excellence (ARNAS Di Cristina-Benfratelli), Palermo, Italy

**Keywords:** Omental pregnancy, nulliparous woman, abdominal tenderness

## Abstract

Pregnancy, the implantation of a fertilized ovum outside the endometrial cavity, occurs in 1.5%-2% of pregnancies. It is one of the major causes (about 6%) of maternal death during the first trimester of pregnancy. The remaining 5% implant in the ovary, peritoneal cavity, within the cervix, and the omental pregnancy is the least common form of abdominal pregnancies. A review of the literature on Medline for the period 1958-2012 reported only 16 cases of omental pregnancy. Here we report a case of primary omental pregnancy in a nulliparous woman. A 24 year-old woman gravid 1, para 0, with lower abdominal pain. Her last menstrual period occurred 8 weeks before the visit. The physical examination revealed abdominal tenderness in the lower quadrants, she was not bleeding. Transvaginal ultrasound showed: a free anechoic/hypoechoic area of 30 x 57 mm in the pouch of Douglas and the endometrium was homogeneus with a thickness of 12 mm and no evidence of gestational sac in the uterine cavity. Laboratory data revealed a normal cell blood count and beta hcg levels of 8047 IU / L. Because of continuing abdominal pain and a diagnosis of ectopic pregnancy a diagnostic laparoscopy was performed, which showed hemoperitoneum. Further inspection of abdominal cavity revealed a bloody lesion that was tenaciously adherent to the omentum, using non traumatic laparoscopic forceps and bipolar scissors we carefully removed a friable mass of about 30 mm from the omental attachments. Histological examination showed the presence of blood clot material mixed with trophoblastic tissue. Ultrasound evaluation and and hCG assessment are important to determine the extrauterine location of the ectopic pregnancy but the early diagnosis of abdominal pregnancy requires also a laparoscopic evaluation and, as our case has highlighted, thorough abdominal exploration especially in the absence of adnexal findings when ectopic pregnancy is highly suspected. Early diagnosis of omental pregnancy is difficult but essential to reduce the high mortality risk for the mother.

## Introduction

Ectopic pregnancy, the implantation of a fertilized ovum outside the endometrial cavity, occurs in 1.5%-2% of pregnancies. It is one of the major causes (about 6%) of maternal death during the first trimester of pregnancy [1]. Nearly 95% of ectopic pregnancies are implanted in one of the segments of the fallopian tubes. The remaining 5% implant in the ovary, peritoneal cavity, within the cervix, and the omental pregnancy is the least common form of abdominal pregnancies [2]. In 1942, Studdiford reported a well-documented case and established criteria for defining all problem cases. This criteria was modified by Friedrich and Rankin in 1968 [3]. A review of the literature on Medline for the period 1958-2012 reported only 16 cases of omental pregnancy. Here we report a case of primary omental pregnancy in a nulliparous woman.

## Patient and observation

**Figure 1:**
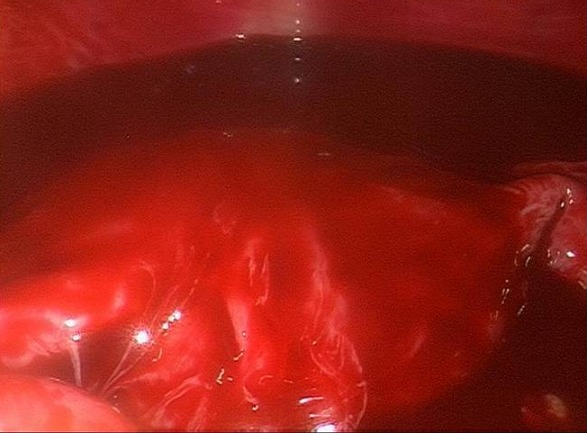
Laparoscopy showed a moderate hemoperitoneum

**Figure 2:**
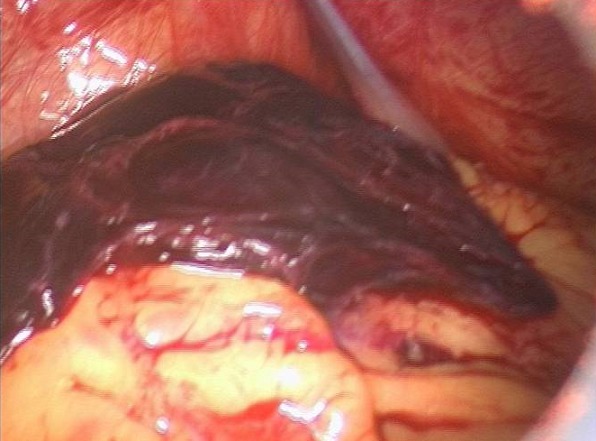
In the surface of omentum was a lesion that seems attributable to blood clot, tenaciously adhered to omental tissue

**Figure 3:**
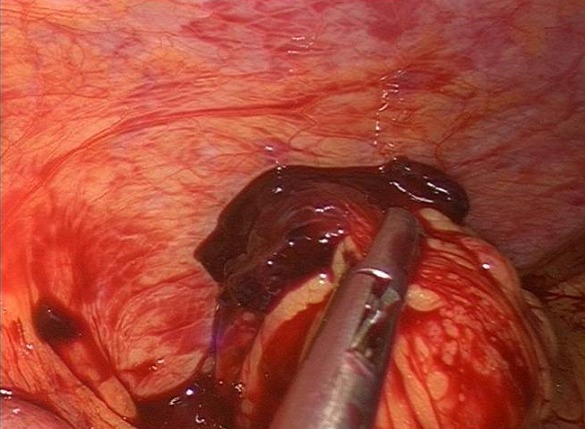
In the surface of omentum was a lesion that seems attributable to blood clot, tenaciously adhered to omental tissue

**Figure 4:**
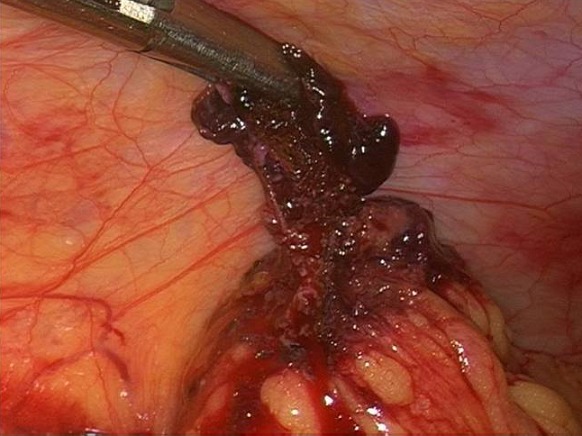
Friable mass of about 30 mm removed by forceps and bipolar scissors

**Figure 5:**
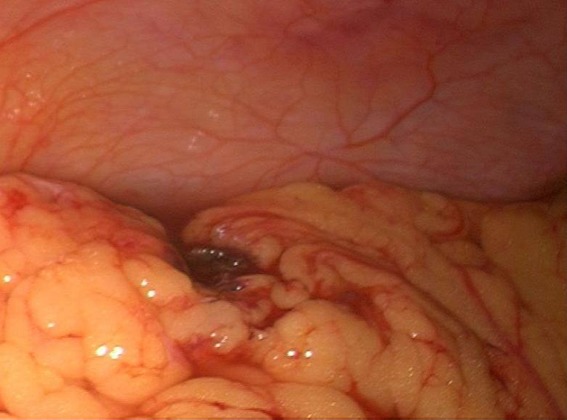
A friable mass of about 30 mm removed by forceps and bipolar scissors

A 24 year-old woman gravid 1, para 0, presented to the Obstetric and Gynecology Department of the Civic Hospital of Palermo with lower abdominal pain. Her last menstrual period occurred 8 weeks before the visit. At the time of presentation, her pulse was 80 beats/min, blood pressure was 100/65 mm HG and temperature was 36.4 °C. The physical examination revealed abdominal tenderness in the lower quadrants, she was not bleeding. Uterus was anteverted, smooth, non tender, not enlarged. The adnexa were not palpable and not tender. Transvaginal ultrasound showed: a free anechoic/hypoechoic area of 30 x 57 mm in the pouch of Douglas, normal adnexa for volume and morphology, in the left ovary there was a cystic lesion (24 x 22 mm), hypoechoic, referable to the corpus luteum, normal uterus and the endometrium was homogeneus with athickness of 12mm and no evidence of gestational sac in the uterine cavity. Laboratory data revealed a normal cell blood count and beta hcg levels of 8047 IU / L. Because of continuing abdominal pain and a diagnosis of ectopic pregnancy a diagnostic laparoscopy was performed, which showed hemoperitoneum of about 210 cc and normal uterus and adnexa (Figure 1). Further inspection of abdominal cavity revealed a bloody lesion that was tenaciously adherent to the omentum (Figure 2, Figure 3). Two additional trocars were inserted and using non traumatic laparoscopic forceps and bipolar scissors we carefully removed a friable mass of about 30 mm (Figure 4, Figure 5) from the omental attachments. Hemostasis was achieved with bipolar coagulation without suturing the omental breach. Within 24 hours the patient was pain free and 2 days after laparoscopy she was discharged with a beta HCG of 600 IU /L. Histological examination showed the presence of blood clot material mixed with trophoblastic tissue.

## Discussion

Towards the end of the 19th century the first diagnostic strategies for ectopic pregnancy were reported, occasionally with successful outcome for the women [4]. At that time the pre-operative diagnosisof a ruptured ectopic pregnancy was false in about 20% of women, while the diagnosis of an un ruptured ectopic pregnancy was virtually impossible [5]. Further technological progress led to the introduction of ultrasonography. Towards the end of the 1960s, ultrasonography was shown to improve thelikeliness of an ectopic pregnancy in the absence of a visualized intra uterine pregnancy. Ultrasounddiagnosis of ectopic pregnancy was hoped to bea non invasive alternative for laparoscopy.

Over the last decades, transvaginal ultrasound has become the first step in the diagnosis of ectopic pregnancy. Between 87% and 99% of tubal pregnancies can now be diagnosed reliably using transvaginal ultrasound. Sensitivity of transvaginal ultrasound as a single test in the diagnosis of ectopic pregnancy is 74% (95% CI: 65.1-81.6) with a specificity of 99.9% (95% CI: 99.8-100) [6]. Approximately 60% of ectopic pregnancies are seen as an in homogeneous mass(“blob sign”) adjacent to the ovary, 20% appear as a hyperechoicring (bagel sign) and 13% have an obvious gestationalsac with a fetal pole, with or without fetal cardiac activity. Only a small proportion of women will have a positive pregnancy test and an in conclusive scan, e.g. no intrauterine or ectopic pregnancy or retained products of conception seen at transvaginal ultrasound. These women are categorized asa pregnancy of unknown location (PUL). This is a descriptive term rather than a pathologic entity. The second important element in the history of diagnosing ectopic pregnancy was measurement of concentrations of human chorionic gonadotropin (hCG). In the 1960s, the production of an immunologic test specific for the beta subunit of hCG in urine led to a clinical application of the pregnancy test. The sensitivity improved from 20,000 IU/L to1, 000 IU/L and the detection time up to five minutes which greatly attributed to its applicability in the diagnosis of early ectopic pregnancy [7].

The importance of combining ultrasound findings with serum hCG concentrations was first recognized by Kadar et al. [8], who introduced the concept of the discriminatory serum hCG zone in 1980. According to this concept, the diagnosis of ectopic pregnancy was most likely whenever an intrauterine pregnancy was not detectable by (abdominal) ultrasound at serum hCG concentrations above a threshold of 6,500 IU/l. The more sensitive and rapid serum hCG tests and the vaginal ultrasound probes with high resolution brought the optimal serum hCG cut-off value of the discriminatory zone concept down to between 1,000 and 2,000 IU/l for women with in conclusive vaginal Ultrasonographic findings [9]. The use of a laparoscope in the diagnosisof ectopic pregnancy was suggested in 1937 by Hope in the United States [10]. From the late 1960s onward, laparoscopy was moreand more used in the diagnostic management of ectopic pregnancy. This technique solved the dilemma of prolonged clinical observation, the risk of performing an unnecessary laparotomy and resulted in an earlier diagnosis of ectopic pregnancy. Laparoscopy remained the most reliable method for diagnosing or excluding ectopic pregnancy until well into the 1980s the national guidelines in the United States and the United Kingdom recommended two diagnostic algorithms for clinical practice [9].

We have found a case report of primary peritoneal pregnancy [11]. Omental implantation of ectopic gestational tissue after surgery for ectopic pregnancy is rare. In our case the patient has never been subjected to surgery. Most ectopic pregnancies are located in the ampullary segment ofthe fallopian tube. However, they may also occur within the interstitial portion of the fallopian tube, in the uterine cervical canal, between the leaves of the broad ligament, in the ovary, within a scar from a cesarean section, or in the abdomen. These unusual ectopic pregnancies are difficult to diagnose and are associated with significant morbidity and mortality [12]. Abdominal pregnancies have been classified as either primary orsecondary. Most abdominal pregnancies originate as tubal or ovarian pregnancies after rupture into the peritoneal cavity, they implant for a second time (secondary abdominal pregnancy) [13]. A small fraction of the reported cases are considered as primary and reflect the three criteria for this condition established by Studdiford [14]: 1) Normal tubes and ovaries with no evidence of recent or remote injury; 2) An absence of any evidence of a utero-peritoneal fistula; 3) The presence of a pregnancy related exclusively to the peritoneal surface eliminates the possibility of a secondary implantation following a primary nidation in the tube. Our case fulfills the three criteria.

In a review of the literature, Friedrich and Rankin found only 24 cases of first trimester pregnancies where the implantation site was only on the peritoneal surface [15]. They proposed modifying Studdiford's criteria to limit acceptable cases as the one of tubal damage that should be clearly evident and the placental site would be easily distinct. Friedrich and Rankin's modifications are as follows: 1) The presence of a pregnancy of less than 12 weeks’ histological gestational age whose trophoblastic attachments are related only to a peritoneal surface; 2) Grossly normal tubes and ovaries; 3) The absence of utero-peritoneal fistula, the diagnosis of peritoneal pregnancy is complex. Ultrasound, when coupled with clinical evaluation, has approximately a 50 percent success rate in the diagnosis [16]. The guidelines for the use of ultrasound to diagnose abdominal pregnancy require: 1) demonstration of a fetus in a gestational sac outside the uterus, or the depiction of an abdominal or pelvic mass identifiable as the uterus separate from the fetus; 2) failure to see a uterine wall between the fetus and urinary bladder; 3) recognition of a close approximation of the fetus to the material abdominal wall; 4) localization of the placenta outside the confines of the uterine cavity. However, the reported diagnostic error rates in different series have ranged from 50 to 90% [17]. A magnetic resonance imaging scan can also be used to confirm the diagnosis of abdominal pregnancy. Laboratory tests, such as abnormally increasing human chorionic gonadotropin, are not sufficiently reliable on their own to make a diagnosis, nor are signs and symptoms such as abdominal pain and tenderness, persistent transverse or oblique lie, or palpable fetal parts. We have also completed a literature review of MEDLINE which showed sixteen cases of primary omental pregnancy between 1958 and 2012 (Table 1). In our case, the only evidence of ectopic pregnancy was the sustained high level of serum beta Hcg, abdominal pain and fluid in the pouch of the Douglas.

**Table 1:** A literature review of MEDLINE showed sixteen cases of primary omental pregnancy between 1958 and 2012

Authors	Years
Cavanagh D.	1958
Friederich M.A.	1968
De Vriesk et al.	1980
Chang et al.	2002
Ozdemir I et al.	2003
Chung Wong W. et al.	2004
Onan MA et al.	2005
Karaer O. et al	2007
Yildizhan R. et al.	2008
Borges da Silva B. et al.	2008
Hornemann A. et al.	2008
Hwa Hong J. et al.	2008
Esin S. et al.	2009
Chopra S. et al.	2009
Seal H.J. et al.	2010
Akthar M.A.	2012

The traditional management of abdominal pregnancy is surgery by laparotomy. Laparoscopic management has not been advocated because the control of hemorrhage can be difficult owing to trophoblastic invasion of retroperitoneal vasculature. Nevertheless, there have been reports of laparoscopic management, which can be associated with a shorter operative time and reduced, blood loss [18]. Because of associated economic and social benefits, the laparoscopic approach should be chosen, except for patients with extensive intraperitoneal bleeding, vascular compromise, or poor visualization of the pelvis at the time of laparoscopy, when laparotomy cannot be avoided. Our patient was stable and the laparoscopic approach proved to be safe and effective. Adjuvant treatment with systemic methotrexate, or through selective arterial embolization, has been suggested to reduce the risk of uncontrolled bleeding from the placental bed and the possibility of persistent trophoblastic tissue [1], which has been reported to occur more often after conservative surgery than radical procedures for ectopic tubal pregnancy. Other precautions have also been proposed to reduce the risk of persistent EP during laparoscopic surgery, including aspiration of all the blood clots and tissue fragments, minimizing the degree of the Trendelenburg position, meticulous extraction of the trophoblastic tissue from the fallopian tube, and using a tissue retrieval bag [19].

In addition, we recommend the use of suction instead of water irrigation at the site of salpingostomy when checking for bleeders, to avoid iatrogenic deposition of residual chorionic villi which weren't removed completely. Gracia et al. [20] also recommended prophylactic MTX after laparoscopic salpingostomy when the surgeon is not sure whether the entire products of conception were removed. Although surgery remains the main stay of treatment for abdominal ectopic pregnancies, there are also case reports of early abdominal pregnancies treated successfully with systemic methotrexate, leading to its resorption without the need for further surgery. Factors that are associated with failure of medical management include initial b-hCG values greater than 5,000 mUI/mL, ultrasound detection of a moderate or large amount of free peritoneal fluid, the presence of fetal cardiac activity, and a pretreatment increase in theb-hCG level of more than 50% over a 48-hour period [1]. We treated our patient with laparoscopic resection of the omental pregnancy and achieved optimal surgical and clinical results without recurrence.

## Conclusion

Early diagnosis of omental pregnancy is difficult but essential to reduce the high mortality risk for the mother. Ultrasound evaluation and hCG assessment are important to determine the extra uterine location of the ectopic pregnancy but the early diagnosis of abdominal pregnancy requires also a laparoscopic evaluation and, as our case has highlighted, thorough abdominal exploration especially in the absence of adnexal findings when ectopic pregnancy is highly suspected. Systemic methotrexate is an alternative non surgical treatment option in women with an ectopic pregnancy and no signs of active bleeding presenting with low initial serum hCG concentrations. Expectant management may be considered in women with low and plateauing serum hCG. Early diagnosis allows therefore, to avoid serious complications and resolve a disease with a high mortality rate.
